# Supplementary figures and images for: Copy Number Variant Detection with Low-Coverage Whole-Genome Sequencing Represents a Viable Alternative to the Conventional Array-CGH
Source: Diagnostics (Basel). 2021 Apr 15;11(4):708. doi: 10.3390/diagnostics11040708 (PMC8071346; doi:10.3390/diagnostics11040708)

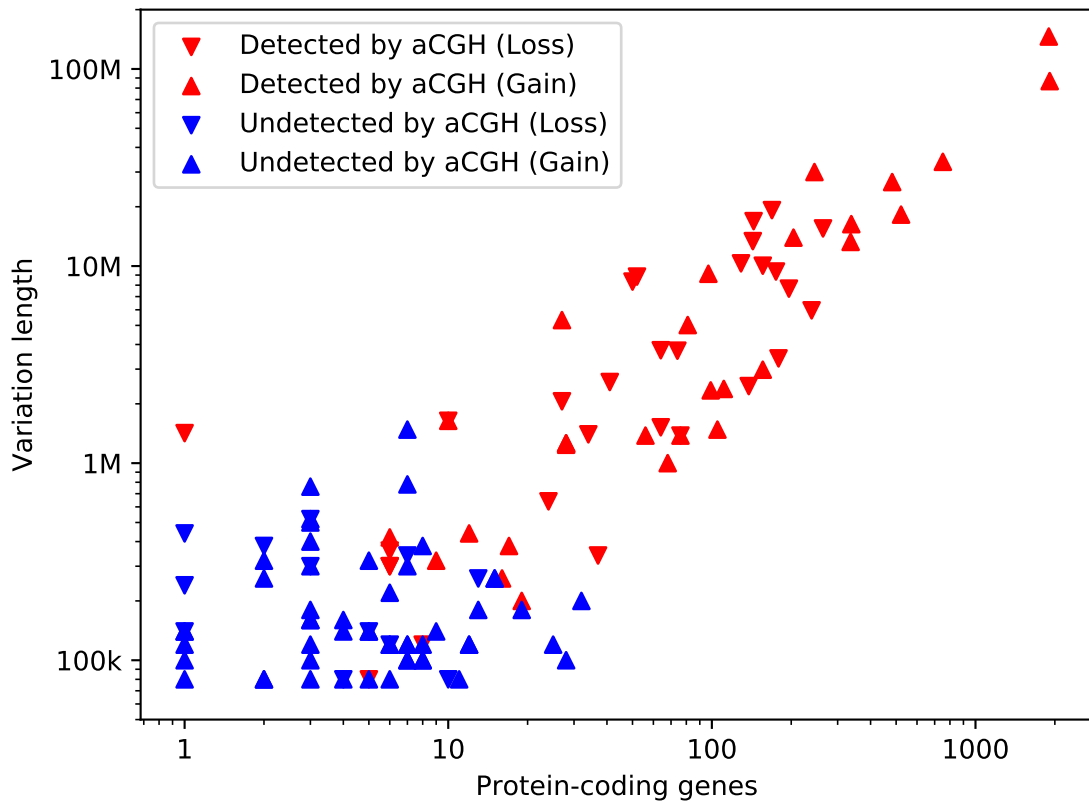

Supplement: Supplementary file 1 [file diagnostics-11-00708-s001.zip › Supplementary Material Figure S1.pdf]

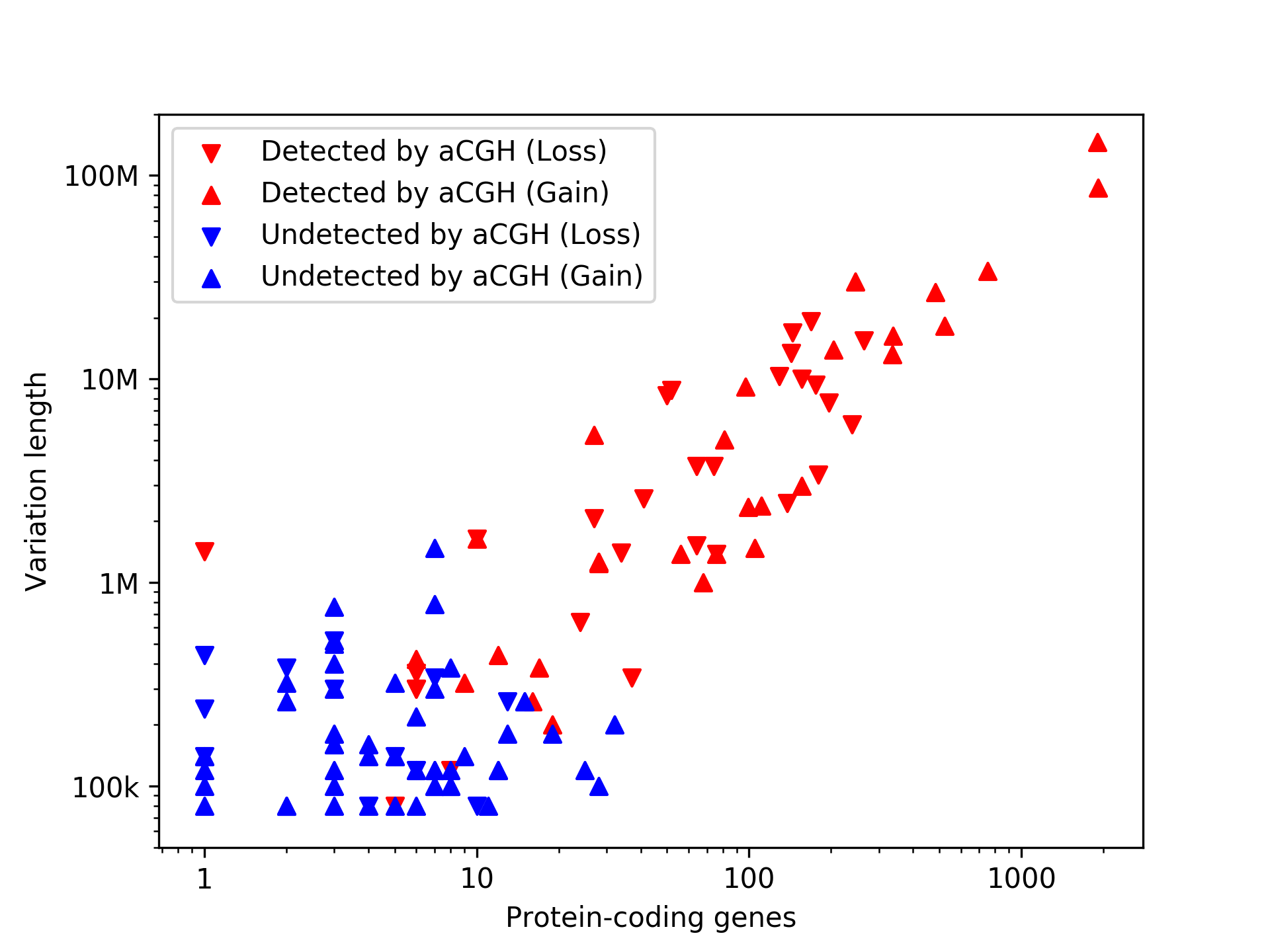

Supplement: Supplementary file 1 [file diagnostics-11-00708-s001.zip › Supplementary Material Figure S1.png]
